# Post-Translational Modification of Human Histone by Wide Tolerance of Acetylation

**DOI:** 10.3390/cells6040034

**Published:** 2017-10-12

**Authors:** Cuiling Li, Han-Pil Choi, Xiaoyue Wang, Fei Wu, Xinjun Chen, Xin Lü, Ruirui Jing, Hoon Ryu, Xingyuan Wang, Kazem M. Azadzoi, Jing-Hua Yang

**Affiliations:** 1 Cancer Research Center, Shandong University School of Medicine, Jinan 250012, China; cuilingli@yahoo.com (C.L.); xywang88@yahoo.com (X.W.); urowufei@gmail.com (F.W.); jam@mail.sdu.edu.cn (X.C.); lvxin121@163.com (X.L.); jingruirui223@163.com (R.J.); 2 Departments of Surgery, Urology, Neurology and Proteomics Laboratory, VA Boston Healthcare System, Boston University School of Medicine, Boston, MA 02130, USA; hpchoi89@gmail.com (H.-P.C.); hoonryu@bu.edu (H.R.); 3 Department of Management Science, Shandong University School of Management, Jinan 250100, China; wangxingyuan@126.com

**Keywords:** post-translational modifications, mass spectrometry, human histones, acetylation, proteomics

## Abstract

Histone acetylation adds an acetyl group on the lysine residue commonly found within the N-terminal tail protruding from the histone core of the nucleosome, and is important for chromosome structure and function in gene transcription and chromatin remodeling. Acetylation may also occur on other residues additional to lysine, but have not been thoroughly investigated at the proteomics level. Here we report a wide tolerance acetylation study mimicking the addition of 42 ± 0.5 Da delta mass modification on undefined amino acid residues of histones by shotgun proteomics using liquid chromatography–tandem mass spectrometry. A multi-blind spectral alignment algorithm with a wide peptide tolerance revealed frequent occurrence of 42 ± 0.5 Da modifications at lysine (K), serine (S) and threonine (T) residues in human histones from kidney tissues. Precision delta mass analysis identified acetylation (42.011 ± 0.004 Da) and trimethylation (42.047 ± 0.002 Da) modifications within the delta mass range. A specific antibody was produced to validate the acetylated T22 of human histone H3 (H3T22ac) by immune assays. Thus, we demonstrated that the wide tolerance acetylation approach identified histone acetylation as well as modification variants commonly associated with acetylation at undefined residues additional to lysine.

## 1. Introduction 

Histone acetylation involves a cellular process that enzymatically transfers an acetyl group from acetyl-Coenzyme A to the epsilon-amino group of lysine residues in histones by “histone acetyltransferases” (HATs). Histone acetylation typically occurs at lysine residues within the histone N-terminal tails to form *N*-acetyl-lysine modifications on the nucleosome surface protruding from the nucleosome’s core. The nucleosomes are linked by histone H1, and the core is an octamer composed of two H2A–H2B dimers and two H3–H4 dimers, which are wrapped around by 145–147 base pairs of double-stranded DNA (dsDNA). The N-terminal tails of histone are rich in lysine residues, so they are positively charged to interact with the negatively charged phosphate groups of DNA on the nucleosome surface to form chromatins in eukaryotic cells. Acetylation of histone lysine residues neutralizes the positive charge and disrupts the interaction between histones and DNA, resulting in the relaxation of condensed chromatins and activation of gene transcription [1,2,3,4]. In contrast, deacetylation by histone deacetylases (HDACs) makes the DNA tightly wrapped around the histone cores, leading to repression of gene transcription [2,3,5]. In addition, acetylation of histone lysine residues recruits the proteins responsible for transcriptional activation to the elongated chromatin marked by acetyl groups [6,7]. Histone acetylation and deacetylation are recognized as major regulatory mechanisms in the activation and repression of gene transcription and regulation of different stages of genetic processes [8]. 

Many acetylated lysine residues are reported within the N-terminal tails of histones at the positions of H2A K5, H2B K2, 12, 15 and 20, H3 K9, 14, 18 and 23, and H4 K5, 8, 12 and 16 [9]. Acetylation in these regions significantly affects interactions with DNA and thus regulates transcription and repression. Lysine acetylation at the histone fold region and C-terminal tails are also reported [10]. For instance, histone H3 K56 in the DNA entry–exit region [11,12,13,14], H3 K115 and H3 K122 in the dyad region [15], and histone H4 K77 and H4 K79 in the DNA–histone interface about 35 base pairs into the nucleosome [16] were shown to be acetylated. Acetylation in these regions affects histone remodeling and assembly to regulate transcriptional activation and DNA replication and repair. 

Nevertheless, acetylation can also occur at the alpha-amino group in the N-termini of histone proteins [17]. The N-terminal acetylation, catalyzed by N-terminal acetyltransferases (NATs), is one of the most common protein modifications in eukaryotes, and acts as a protein degradation signal, an inhibitor of endoplasmic reticulum (ER) translocation, and a mediator of protein complex formation [18]. Markedly, acetylation could also occur with hydroxyl groups to form *O*-acetyl ester modifications, theoretically on the side chains of serine, threonine and tyrosine residues. In fact, *O*-acetylserine is an intermediate in the biosynthesis of cysteine in bacteria and plants, and it is biosynthesized by the enzyme serine transacetylase [19]. In eukaryotes, however, the counterpart of serine transacetylase is not yet identified. During *Yersinia pestis* infection, the deadly disease called bubonic plague, the serine and threonine residues of human proteins are indeed acetylated by the *Yersinia* effector protein YopJ [20,21]. The bacterial YopJ protein acts as an acetyltransferase that binds and acetylates serine and threonine residues in the activation loops of several kinases, including mitogen-activated protein kinase kinases (MAPKKs) and IκB kinase β (IKKβ). Thus, YopJ inhibits their phosphorylation and consequently their activation [20,21].

Mass spectrometry (MS)-based proteomics has led to the discovery of a large number of new histone and non-histone post-translational modifications (PTMs), which can be detected by PTM-related diagnostic mass shifts of fragment ions in MS/MS spectra. Either *N*-acetylation or *O*-acetylation increases the molecular weight of the modified amino acid by 42.01057 Dalton. The current technology of mass spectrometry to identify acetylation needs to determine the peptide masses (MS) and their collision break down fragments (MS/MS) and then compare with the theoretical protein sequences. The delta mass of 42.01057 Dalton is a typical mark for an acetyl group in a peptide with known sequence. 

This study aimed at setting up a wide tolerance acetylation approach to identify *N*- or *O*-acetylation at undefined amino acid residues additional to lysine on human histones using shotgun proteomics. Wide tolerance acetylation search followed by precision delta mass analysis and restricted delta mass search revealed frequent acetylation of human histone proteins at serine and threonine residues in addition to lysine, including putative new acetylation sites on human histones. 

## 2. Materials and Methods

### 2.1. Tissue Samples and Cell Culture 

Human kidney tissue samples (morphologically normal kidney cortex) adjacent to kidney cancer tissues (clear cell renal cell carcinoma) were collected from 12 cancer patients with nephrectomy in full compliance with Institutional Ethics Review Board’s guidance at the Department of Urology, Shandong Provincial Hospital Affiliated to Shandong University. All procedures were consistent with the National Institutes of Health Guide and approved by the institutional board with patients’ written consent. This study was evaluated and approved by the Medical Ethics Committee of Medical School Shandong University. The kidney tissues were excised immediately following nephrectomy, cut into small blocks (~100 mg), rinsed with ice-cold phosphate-buffered saline (PBS), snap-frozen in liquid nitrogen, and stored at −80 °C until protein extraction. For cell culture, HEK293T and HeLa cells were grown in Dulbecco’s Modified Eagle Medium (DMEM) supplemented with 10% fetal bovine serum (FBS) and 1% penicillin/streptomycin.

### 2.2. Histone Protein Extraction 

Histone proteins were isolated from the human kidney tissues by acid extraction procedure [22]. Briefly, nuclei were obtained by homogenizing kidney tissues in hypotonic lysis buffer (10 mM Tris-Cl pH 8.0, 1 mM KCl, 1.5 mM MgCl_2_, 1 mM dithiothreitol) with protease inhibitor cocktail and phosphatase inhibitor cocktail (Roche), and histones were isolated from nuclei by extraction with 0.4 N H_2_SO_4_. For cell culture, cells were collected and lysed in radio immuno precipitation assay (RIPA) lysis buffer (Millipore) with protease inhibitor cocktail (Roche). The cell lysates were centrifuged, and the supernatants were collected and stored at −80 °C until analysis. 

### 2.3. Sodium Dodecyl Sulfate Polyacrylamide Gel Electrophoresis (SDS-PAGE) and In-Gel Digestion 

Combined histone proteins (~200 μg) extracted from 12 different kidney tissues were separated on 12% sodium dodecyl sulfate polyacrylamide gel electrophoresis (SDS-PAGE) gel. Histones H1, H2A/B/H3, and H4 were separately excised from SDS-PAGE gel and digested, in-gel, with trypsin. To ensure the coverage for the lysine-rich region, partially digested histone peptides were recovered from the supernatant of in-gel digestion mixture at 1, 4, 12, and 24 h. The partially digested peptides at different time points were pooled and desalted by C18 ZipTip (Millipore, Billerica, MA, USA). 

### 2.4. Liquid Chromatography–Tandem Mass Spectrometry (LC–MS/MS) 

The extracted and desalted peptides were subjected to peptide fractionation by liquid chromatography on an EASY-nLC 1000 system (Thermo Scientific, San Jose, CA, USA) equipped with a long C18 column (300 × ϕ 0.075 mm, 3 μm particles). Samples were fractionated with an optimized 120 min linear gradient of 5–35% acetonitrile/0.1% formic acid at a flow rate of 250 nL/min. The MS and MS/MS spectra were acquired by a LTQ-Orbitrap Elite mass spectrometer (Thermo Scientific) in a data-dependent mode, in which MS/MS fragmentation of the 20 most intense peaks were acquired for every full MS scan. The acquired MS/MS spectra were searched against the human histone database using MASCOT or SEQUEST. The human histone database was built from UniProtKB (name, histone; keyword, chromosome [KW-0158]; organism, Homo sapiens (human) [9606]; date, 13 January 2015). Percolator was used to calculate the false discovery rate (FDR) of each peptide. Trypsin (full cleavage) was specified as cleavage enzyme allowing up to two missing cleavages. MS/MS spectra were searched with a maximum allowed deviation of 10 ppm for the precursor mass and 0.6 Da for fragment masses. Oxidation (+15.99492 Da) of methionine, acetylation (+42.01057 Da) of lysine, serine and threonine, trimethylation (+42.04695 Da) of lysine, serine and threonine were selected as dynamic modification, cysteine carbamidomethylation (+57.02146 Da) was selected as static modification, and the FDR was 1%. 

### 2.5. Workflow of Wide Tolerance Acetylation Analysis 

This workflow was designed to identify acetylation modifications with wide tolerance on undefined amino acid residues additional to lysine. To this end, unrestricted modification search was conducted using a multi-blind spectral alignment algorithm (MODa) [23] to identify any possible modified amino acids with a delta mass of 42 ± 0.5 Da (to find out peptides with 42 ± 0.5 Da PTM within a background of peptides that could be affected also by other PTMs), while featuring less computation time in comparison with other approaches [24]. The outcomes would include *N*- or *O*-acetylation, amino acid substitution, and new modifications with the delta mass within 42 ± 0.5 Da range. Specifically, the acquired MS/MS datasets were searched against the human histone database described in previous section by MODa with the following parameters: the peptide mass tolerances, ±0.5 Da; fragment ion tolerance, ±0.2 Da; missed cleavage by trypsin, 2; multi-mod mode which allowed arbitrary number of modifications per peptide. Next, the identified delta masses within the 42 ± 0.5 Da range were clustered on their frequencies and the most frequently occurred delta masses were selected as the potential modifications. The amino acid residues with potential delta masses were further confirmed as dynamic modifications by the sophisticated search algorithm SEQUEST at FDR 1% using target-decoy approach. All modification site assignments were confirmed by manual spectrum interpretation. 

### 2.6. Production of Anti-H3T22ac Antibody 

The specific anti-H3T22ac antibody was generated and purified by Sangon (Shanghai, China). Peptide KQLATKAAR (H3T22) and KQLATacKAAR (H3T22ac) with acetyl-threonine were synthesized; the acetylated peptide was conjugated to bovine serum albumin (BSA) as an antigen. The antigen was injected to New Zealand white rabbits until reaching serum titer of more than 1:10,000. The antiserum was first cleaned with immobilized peptide KQLATKAAR to remove unmodified peptides and then purified by immobilized peptide KQLATacKAAR. 

### 2.7. Western Blot Analysis 

About 50 μg of cell lysates from HEK 293T and HeLa were separated on SDS-PAGE gel. After electrophoresis, proteins were transferred to nitrocellulose membranes. The membranes were blocked and probed with primary antibodies against H3 (Abcam) and H3T22ac (Sangon, Shanghai, China). Proteins were detected using fluorescent conjugated secondary antibody (Goat anti-Rabbit Alexa Fluor^®^ 488, Abcam, Cambridge, MA, USA).

## 3. Results and Discussion

### 3.1. High Peptide Coverage of Human Histones 

**Figure 1:**
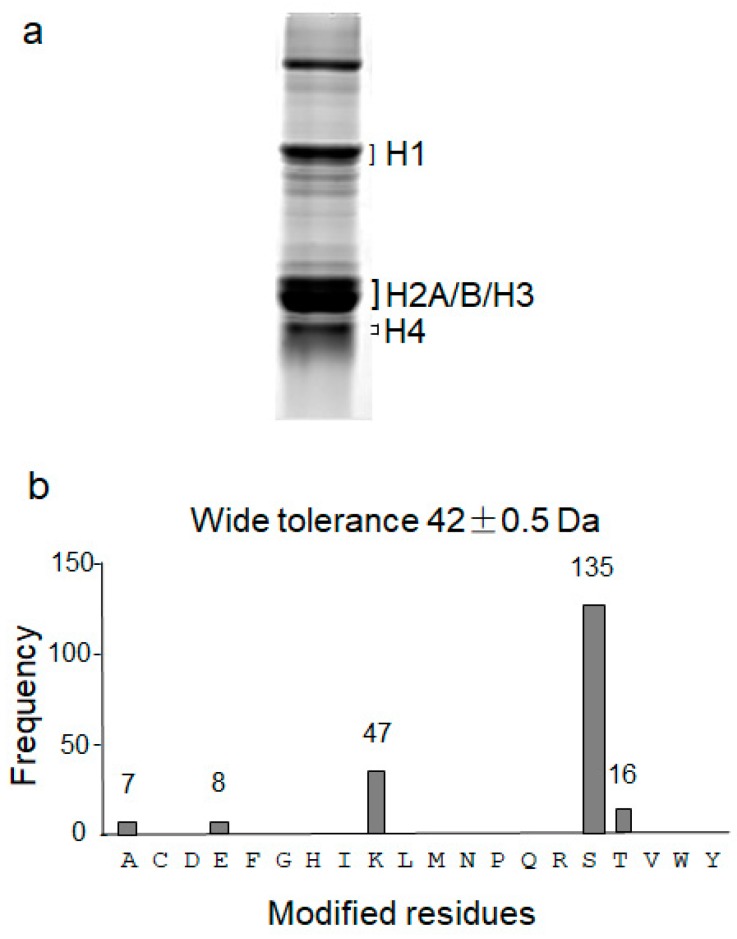
Wide tolerance acetylation (42 ± 0.5 Da) analysis at different amino acid residues of human histones. (**a**) Sodium dodecyl sulfate polyacrylamide gel electrophoresis (SDS-PAGE) of histone proteins from human kidney tissues. Histone proteins isolated from human kidney tissues were resolved by 12% SDS-PAGE and stained with Coomassie Brilliant Blue. The excised regions of the linker histone H1 and the core histones H2A/B/H3 and H4 were marked. (**b**) Frequency of wide tolerance acetylation (42 ± 0.5 Da). The acquired MS/MS spectra of histones were searched by a multi-blind spectral alignment algorithm (MODa) mimicking addition of 42 ± 0.5 Da delta mass on undefined amino acid residues against the human histone database. Frequency of the modified peptides at each amino acid was shown as indicated.

By trypsin digestion, the peptide coverage for histone proteins was usually low due to the abundance of lysine and arginine residues in the sequences. Chemical protection of the lysine residues by partial propionylation was previously performed to partially block trypsin digestion [25]. In this study, the shotgun proteomic approach was improved to increase the peptide coverage for histone proteins. Firstly, histones were enriched by the acid extraction procedure [22] from human kidney tissues. The enriched histone proteins were separated with 12% sodium dodecyl sulfate polyacrylamide gel electrophoresis (SDS-PAGE) and the protein bands corresponding to histone H1, H2A/B/H3 and H4 were excised from the gel (Figure 1a). Secondly, slices from multiple gels were pooled collectively to ensure sample size greater than 10 µg for each histone, which were partially digested, in-gel, with trypsin for 1, 4, 12, and 24 h. The partially digested fragments were pooled to increase the peptide coverage. Typically, over 2 µg of peptides were loaded to a long C18 reverse-phase liquid chromatography column (300 × ϕ 0.075 mm, 3 μm) and eluted with a step/linear gradient optimized for MS and MS/MS data acquisition. The MS/MS spectra were searched against the human histone database using MASCOT and SEQUEST. A total of 158 (88%), 108 (97%), 54 (96%) and 22 (90%) peptides were identified for human histone H1, H2A/B, H3 and H4, respectively (Table S1).

### 3.2. Wide Tolerance Acetylation Analysis of Human Histones at Undefined Amino Acid Residues 

**Figure 2:**
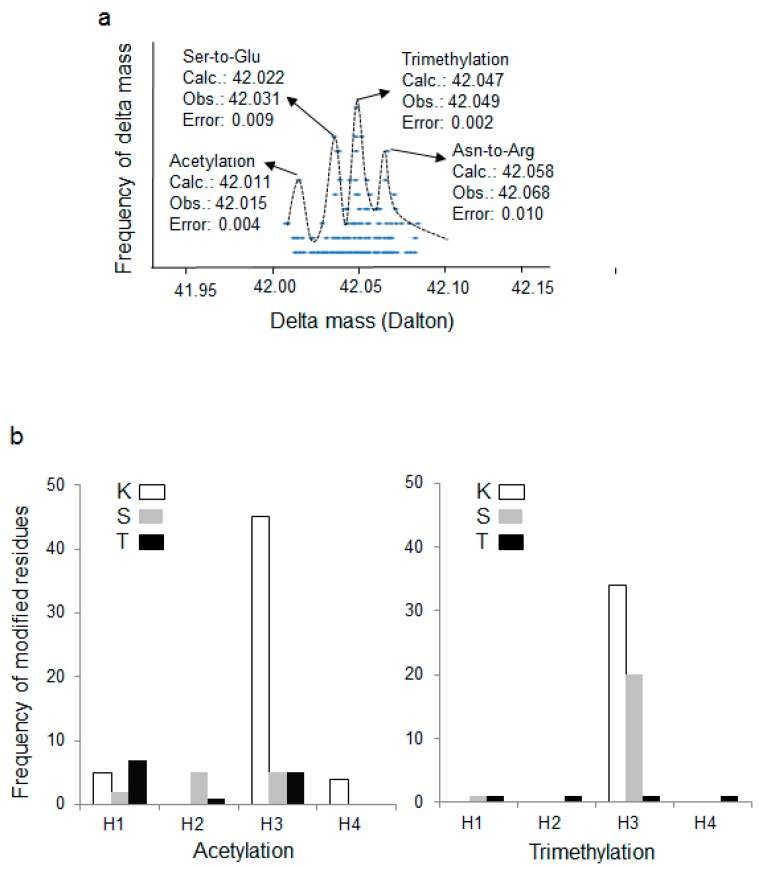
Clustering of the accurate delta masses at different residues of histones. (**a**) Frequency was plotted against their accurate delta masses. The calculated delta masses (Calc.) were from Unimod (http://www.unimod.org) and the observed delta masses (Obs.) were determined by the peaks considering the error range <10 ppm and the total frequency >20; (**b**) Acetylation and trimethylation at different residues of histones H1, H2 (H2A/B), H3 and H4 were confirmed by restricted search using SEQUEST. K, S and T, three amino acid residues as indicated.

To identify possible acetylation modifications without restriction of the modified amino acid residues, a wide tolerance acetylation using MODa [23] was performed. MODa is a “multi-blind” spectral alignment algorithm, enabling fast unrestrictive PTM searches with no limitation on the number of variable modifications per peptide. In this approach, wide tolerance modification search mimicking addition of 42 ± 0.5 Da delta mass on undefined amino acid residues was performed by searching the acquired MS/MS spectra against the human histone database. In total, 213 modified histone peptides were found with the 42 ± 0.5 Da modifications at lysine (Lys or K), serine (Ser or S), threonine (Thr or T), alanine (Ala or A) and glutamic acid (Glu or E) (Figure 1b). Among them, 47 matched peptides were modified at the Lys residue, which included the well-studied Lys-acetylation. The mostly identified 42 ± 0.5 Da modifications were found at Ser (S, 135). The 42 ± 0.5 Da modifications were observed less frequently at Ala (A, 7) and Glu (E, 8). Notably, although wide peptide tolerance was used in unrestricted modification search, the modified residues were determined with high accuracies by LTQ Orbitrap Elite mass spectrometer. Thus, the accurate delta masses were analyzed against their frequencies to explore whether they were clustered into certain modifications with acceptable errors (<10 ppm or ~0.01 Da) (Figure 2a). Indeed, majority of the delta masses were located within the mass range between 42.00 and 42.10 Da, which were roughly clustered to the following two modifications: (1) acetylation with the observed delta mass of 42.015 Da and calculated delta mass of 42.01057 Da, and (2) trimethylation with the observed delta mass of 42.049 Da and calculated delta mass of 42.04695 Da. Since most delta masses were observed at Lys, Ser, and Thr (Figure 1b) and in view of chemical restrictions that acetylation might occur at the Lys, Ser and Thr residues and trimethylation might occur at the Lys residue, our data suggested that K-, S-, and T-acetylation and K-trimethylation might be further tested on histones from human kidney. Note that acetylation and trimethylation at the N-terminal amino group were not excluded that might cause identification of modification at any residues because of protein N-terminal methionine excision [26,27]. In addition, a few amino acid substitutions were also fallen in this delta mass range, including (1) Gly-to-Val or Ala-to-Leu substitution (observed delta mass of 42.049 Da, same as trimethylation), (2) Ser-to-Glu substitution (observed delta mass of 42.031 Da), and (3) Asn-to-Arg substitution (observed delta mass of 42.068 Da).

### 3.3. Putative New Acetylation Modifications on Human Histones by Restricted Delta Mass Search 

**Figure 3:**
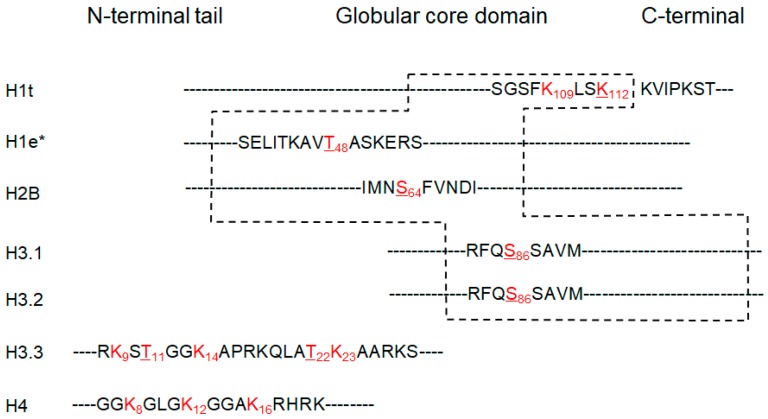
Human histone acetylation sites identified by the wide tolerance acetylation workflow. A diagram showing sites of histone acetylation identified by the wide tolerance acetylation workflow. Red, acetylation sites; underlined, new in human histones; dotted box, the globular core domain; subscript, position of the amino acid residues; *, liver histone H1e.

To confirm the modifications, the acquired MS/MS data were further searched with more sophisticated algorithm using SEQUEST. Restricted acetylation (+42.011 ± 0.01) and trimethylation (+42.047 ± 0.01) with accurate delta masses at the Lys, Ser and Thr residues were searched as dynamic modifications. A total of 138 spectra of the modified residues were identified, among which 79 (57%) were acetylation and 59 (43%) were trimethylation (Figure 2b and Table S2). For the acetylated residues, 54 occurred at Lys, 12 at Ser and 13 at Thr. For the trimethylated residues, however, 34 occurred at Lys, 21 at Ser and 4 at Thr. The distribution of acetylated and trimethylated residues were selective on different histones as they were found predominantly on histone H3. T-acetylation was preferably found on H1 whereas K-acetylation on H3, and S-acetylation on H2 (H2B) and H3 (Figure 2b). Notably, the core histone protein typically consists of an N-terminal tail, a globular core including a central histone-fold domain, and a C-terminal tail. The previously identified histone acetylation modifications were commonly mapped to N-terminal tails. In this study, 6 putative new acetylation sites on human histones were identified from human kidney tissues (Figure 3 and Figure S1). Interestingly, among these sites, only two sites were mapped to the N-terminal tail domain, while most of sites (4) were mapped to the globular core domains. One site of serine was found to be acetylated on histone H2B (S64), and 1 on H3 isoforms (S86 on H3.1 and H3.2). One threonine was found to be acetylated on liver histone H1e (T48), and 2 on H3.3 (T11, T22). In addition, one site of lysine acetylation was identified on H1t (K112). The putative new sites of serine/threonine acetylation, a modification that can prevent/compete the phosphorylation of these residues [20,21], potentially play key functions in chromatin remodeling. They might have significant impact on the functions of histone phosphorylation in DNA repair, transcription and chromatin compaction during cell division and apoptosis [28]. Acetylation of lysine residues in the globular domain of H3 (H3K64ac and H3K122ac) marked active gene promoters and also a subset of active enhancers [29]. Furthermore, in *Saccharomyces cerevisiae,* histone H3 K56 acetylation (globular domain) at the entry–exit gate enabled recruitment of the SWI/SNF (SWItch/Sucrose Non-Fermentable) nucleosome remodeling complex and so regulated gene activity [12]. In view of those findings, the putative new sites of acetylation which were mapped to the globular core domain might have important biological functions for gene expression.

### 3.4. Validation of T-Acetylation in the N-Terminal Tail of Human Histone H3 (H3T22ac) by Immune Assays 

**Figure 4:**
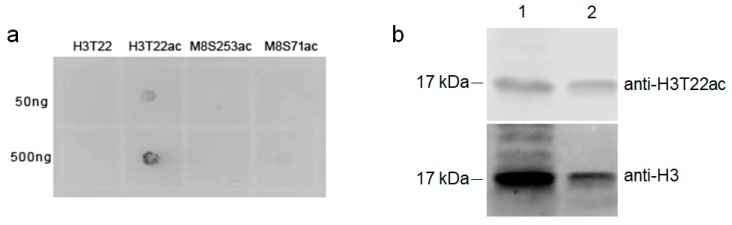
Validation of the acetyl-T22 in human histone H3 by immune assays. (**a**) Specificity of the anti-H3T22ac antibody was demonstrated by dot blot assay using four synthetic peptides: H3T22, un-acetylated peptide KQLATKAAR; H3T22ac, acetylated peptide KQLATacKAAR; M8S253ac, acetylated peptide KSacPLTEPNFENKC; M8S71ac, acetylated peptide TSacITPSSQDICRICHCEGDC. 50 and 500 ng of the peptides were used as indicated; (**b**) Detection of H3T22ac in human histones from different cell lysates. HEK293T (lane 1) and HeLa (lane 2) cell lysates were resolved by SDS-PAGE and detected by western blot assay using the antibodies against H3 (bottom) and H3T22ac (top).

To validate the wide tolerance acetylation workflow, a specific antibody was produced and utilized to confirm the identified modifications. In this study, the acetylation at threonine 22 of human histone H3.3 (H3T22) was selected and the modified peptide KQLATacKAAR (H3T22ac) was chemically synthesized. Notably, H3T22 was a conserved site in human histone H3 variants including H3.1, H3.2, H3.3, H3t, H3.X and H3.Y [30]. There were plenty of PTMs reported near H3T22 such as arginine 17 (R17), lysine 18 (K18), lysine 23 (K23) and lysine 27 (K27). The acetylation of K18 and K23 was found to regulate the activity of coactivator-associated arginine methyltransferase-1 (CARM1) to methylate R17 [31,32]. Recently, acetylation of H3S22 from *Saccharomyces cerevisiae* and H3T22 from *Drosophila melanogaster* was reported [33]. To confirm acetylation of human histone H3T22, a polyclonal antibody recognizing the acetylated human histone H3T22 (H3T22ac) was produced and affinity purified using the modified peptide KQLATacKAAR. The anti-H3T22ac antibody selectively recognized a peptide with the acetylated residue (H3T22ac), but did not detect the same peptide without the acetylated residue (H3T22), or different peptides with acetylated serine at different sites (Figure 4a), confirming the specificity of the antibody. Finally, the anti-H3T22ac antibody was subsequently used to detect the acetylated H3T22ac in HEK293T and HeLa cell lysates by western blot assay (Figure 4b). The anti-H3 antibody was used to show the presence and the position of human histone H3. Positive signals were detected at the same position by the anti-H3T22ac antibody, indicating the presence of the acetylated threonine 22 on human histone H3.

## 4. Conclusions

In this study, a wide tolerance acetylation workflow was used to identify new modifications mimicking the addition of 42 ± 0.5 Da delta mass at undefined amino acid residues of human histones. Shotgun proteomics using liquid chromatography–tandem mass spectrometry (LC–MS/MS) followed with a multi-blind spectral alignment algorithm using a wide delta mass tolerance of 42 ± 0.5 Da modifications revealed a frequent occurrence of 42 ± 0.5 Da modifications at serine, threonine, as well as lysine residues. Accurate delta mass clustering analysis and restricted delta mass search confirmed K-, S- and T-acetylation preferably on human histone H3 (K- and S-acetylation) and on H1 (T-acetylation). Additionally, high frequency of K-trimethylation was also observed on histone H3. A few amino acid substitutions, including Gly-to-Val, Ala-to-Leu, Ser-to-Glu and Asn-to-Arg, were also fallen within the delta mass range. Thus, this workflow was able to identify histone acetylation generally at the residues additional to lysine as well as other modification variants commonly associated with wide tolerance acetylation. Sequentially, K-, S- and T-acetylation of histones could be further validated by immune assays, exemplified by production of specific antibody against the acetylated H3T22ac. In support, S- and T-acetylation was recently reported to be present in histones from mouse brain tissues [34] and human cells (HeLa and HEK 293) [33]. Our study provided an alternative workflow and identified putative new acetylation sites at the serine and threonine residues of histone proteins from human kidney tissues. Future work will be directed at determining the possible role of these modifications in the regulation of histone structure and function.

## Supplementary Materials

The following are available online at www.mdpi.com/2073-4409/6/4/34/s1. Table S1: LC–MS/MS coverage; Table S2: List of peptides with histone acetylation/trimethylation identified in this study and summary of frequency of modified residues; Figure S1: MS/MS spectra of putative new sites of human histone acetylation identified in this study.
