# Supplementary material for: Post-Translational Modification of Human Histone by Wide Tolerance of Acetylation
Source: Cells. 2017 Oct 12;6(4):34. doi: 10.3390/cells6040034 (PMC5753069; doi:10.3390/cells6040034)
Supplement: Supplementary file 1 [file cells-06-00034-s001.zip › Fig_S1_Revised.pptx]

## Slide 1
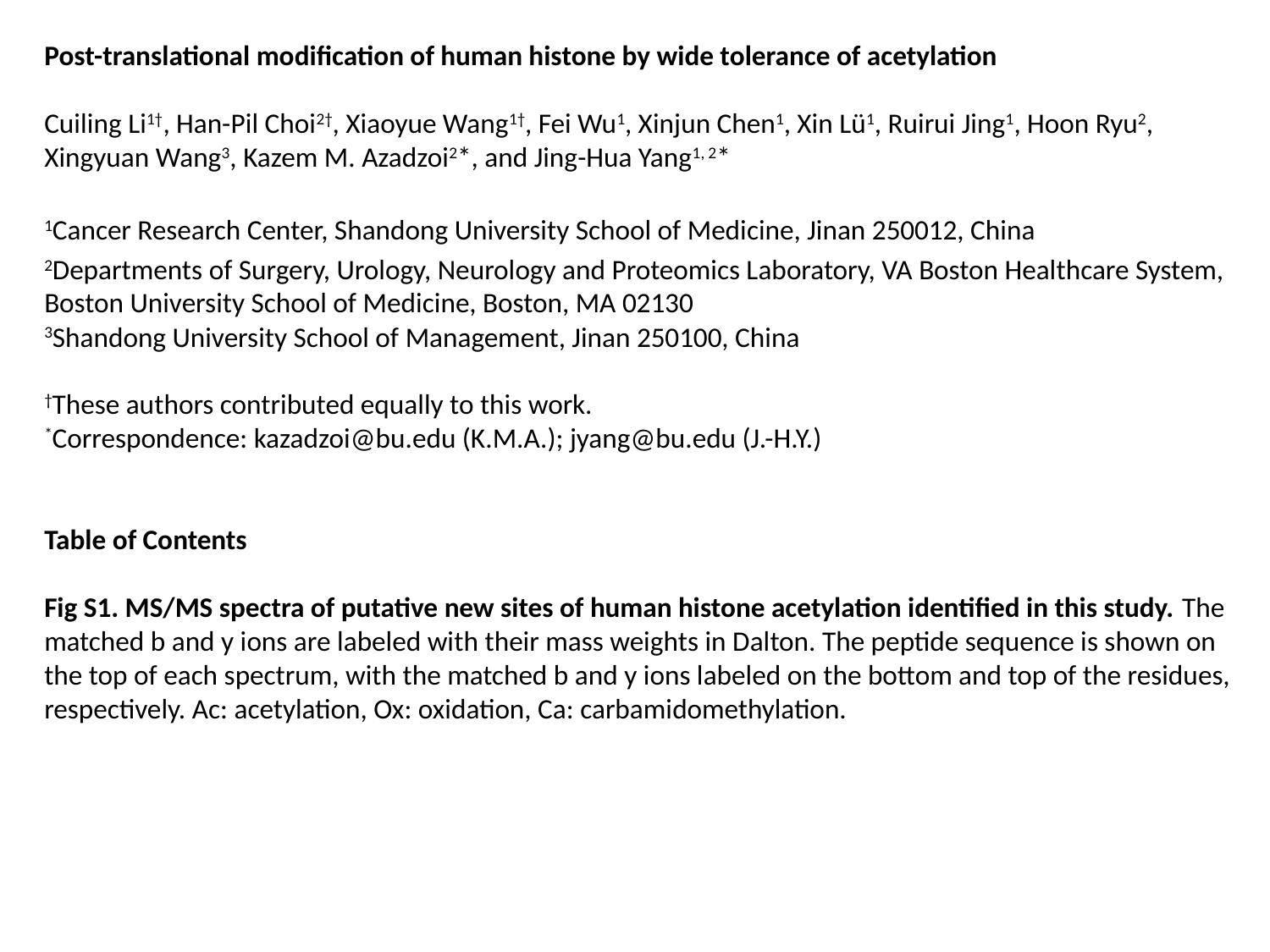

Post-translational modification of human histone by wide tolerance of acetylation
Cuiling Li1†, Han-Pil Choi2†, Xiaoyue Wang1†, Fei Wu1, Xinjun Chen1, Xin Lü1, Ruirui Jing1, Hoon Ryu2, Xingyuan Wang3, Kazem M. Azadzoi2*, and Jing-Hua Yang1, 2*
1Cancer Research Center, Shandong University School of Medicine, Jinan 250012, China
2Departments of Surgery, Urology, Neurology and Proteomics Laboratory, VA Boston Healthcare System, Boston University School of Medicine, Boston, MA 02130
3Shandong University School of Management, Jinan 250100, China
†These authors contributed equally to this work.
*Correspondence: kazadzoi@bu.edu (K.M.A.); jyang@bu.edu (J.-H.Y.)
Table of Contents
Fig S1. MS/MS spectra of putative new sites of human histone acetylation identified in this study. The matched b and y ions are labeled with their mass weights in Dalton. The peptide sequence is shown on the top of each spectrum, with the matched b and y ions labeled on the bottom and top of the residues, respectively. Ac: acetylation, Ox: oxidation, Ca: carbamidomethylation.

## Slide 2
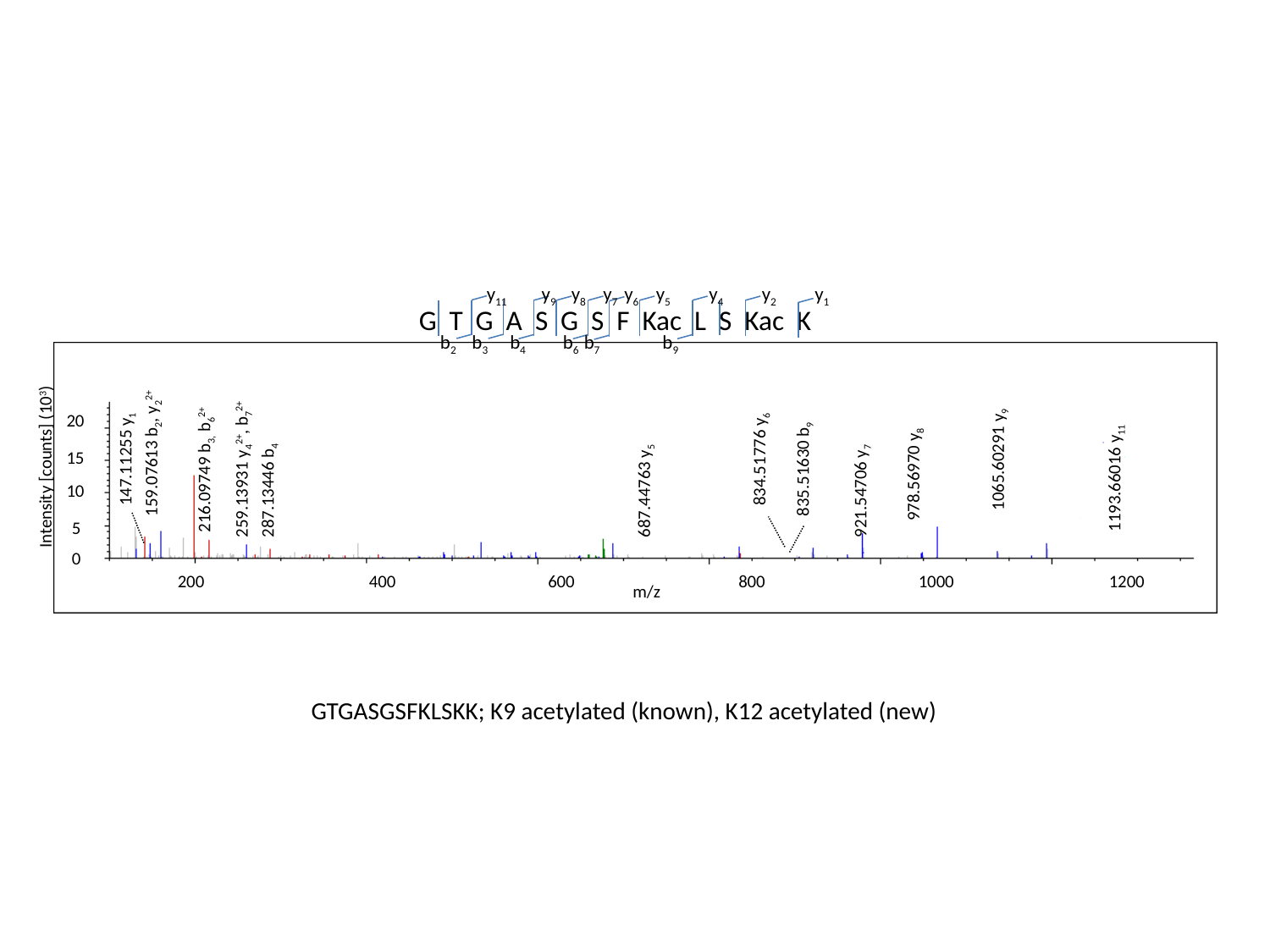

y11
y9
y8
y7
y6
y5
y4
y2
y1
 G T G A S G S F Kac L S Kac K
b2
b3
b4
b6
b7
b9
Intensity [counts] (103)
159.07613 b2, y22+
216.09749 b3, b62+
259.13931 y42+, b72+
834.51776 y6
147.11255 y1
1065.60291 y9
835.51630 b9
978.56970 y8
1193.66016 y11
20
687.44763 y5
287.13446 b4
921.54706 y7
15
10
5
0
200
400
600
800
1000
1200
m/z
GTGASGSFKLSKK; K9 acetylated (known), K12 acetylated (new)

## Slide 3
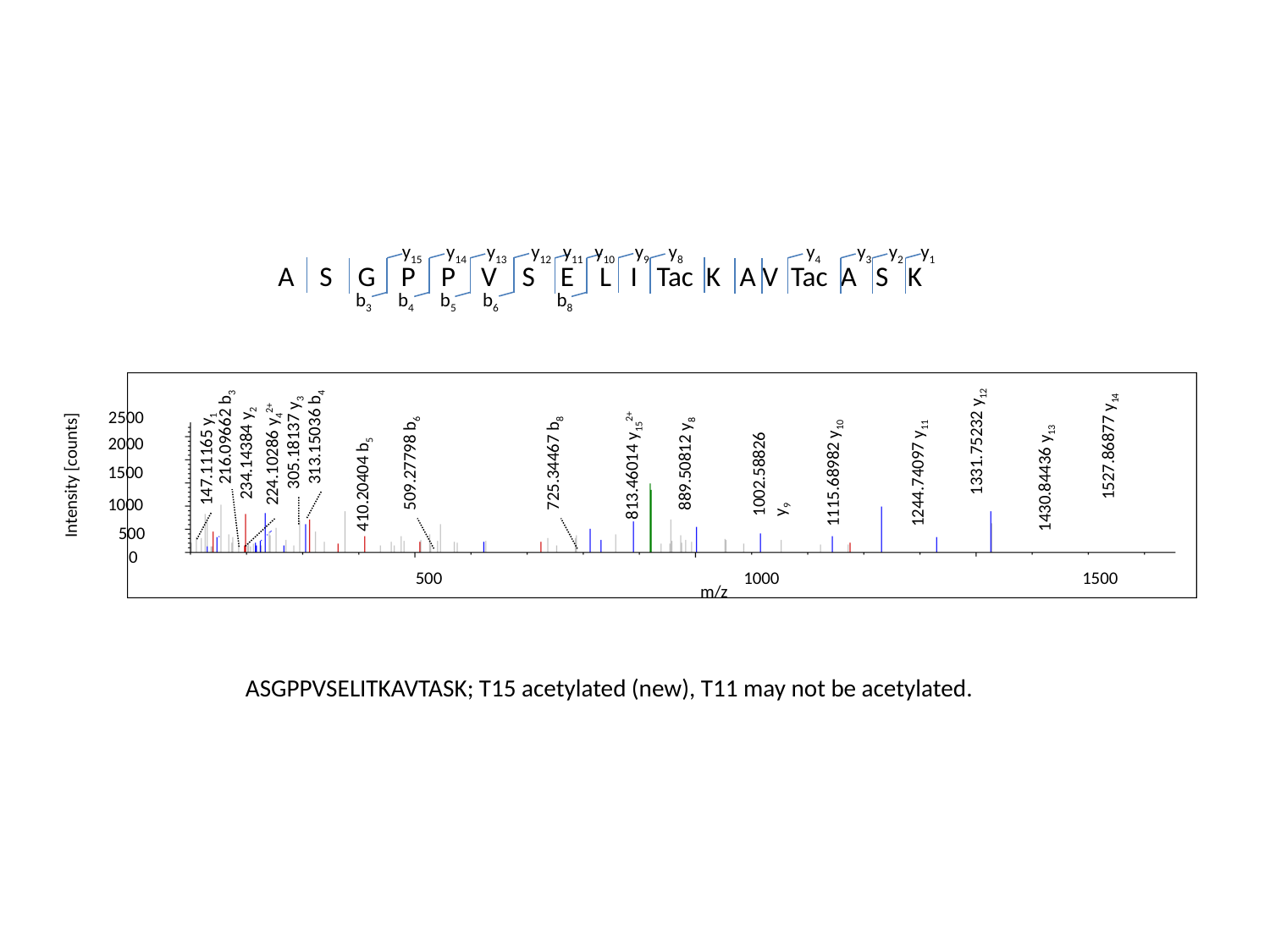

y15
y14
y13
y12
y11
y10
y9
y8
y4
y3
y2
y1
 A S G P P V S E L I Tac K A V Tac A S K
b3
b4
b5
b6
b8
313.15036 b4
305.18137 y3
216.09662 b3
234.14384 y2
1331.75232 y12
147.11165 y1
224.10286 y42+
509.27798 b6
725.34467 b8
1527.86877 y14
Intensity [counts]
813.46014 y152+
2500
410.20404 b5
889.50812 y8
1002.58826 y9
1115.68982 y10
1244.74097 y11
1430.84436 y13
2000
1500
1000
500
0
500
1000
1500
m/z
ASGPPVSELITKAVTASK; T15 acetylated (new), T11 may not be acetylated.

## Slide 4
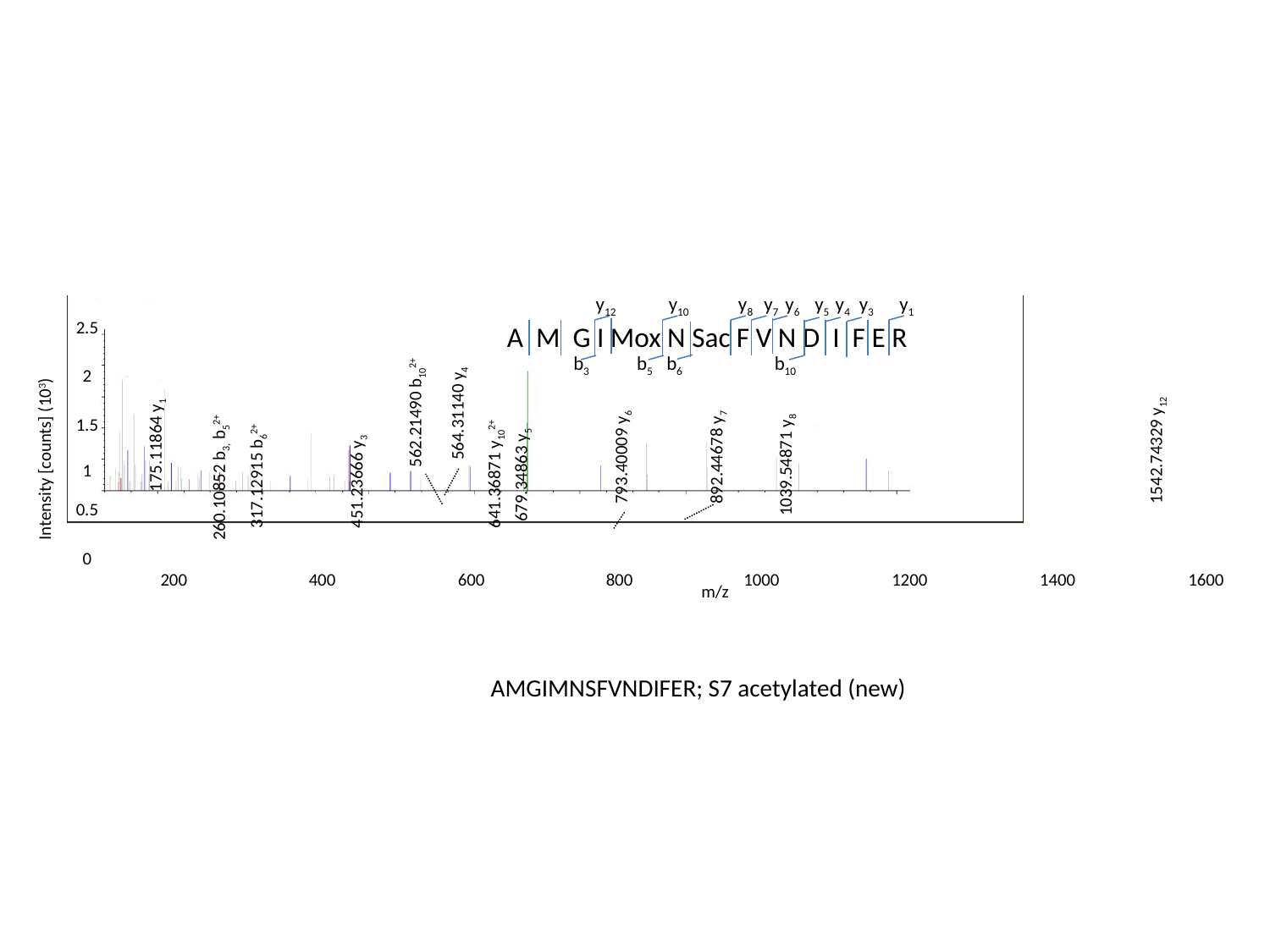

y8
y7
y6
y3
y1
b3
b6
 A M G I Mox N Sac F V N D I F E R
y5
y4
b5
y12
y10
b10
2.5
562.21490 b102+
564.31140 y4
Intensity [counts] (103)
1542.74329 y12
2
317.12915 b62+
175.11864 y1
641.36871 y102+
793.40009 y6
892.44678 y7
260.10852 b3, b52+
1039.54871 y8
679.34863 y5
1.5
451.23666 y3
1
0.5
0
200
400
600
800
1000
1200
1400
1600
m/z
AMGIMNSFVNDIFER; S7 acetylated (new)

## Slide 5
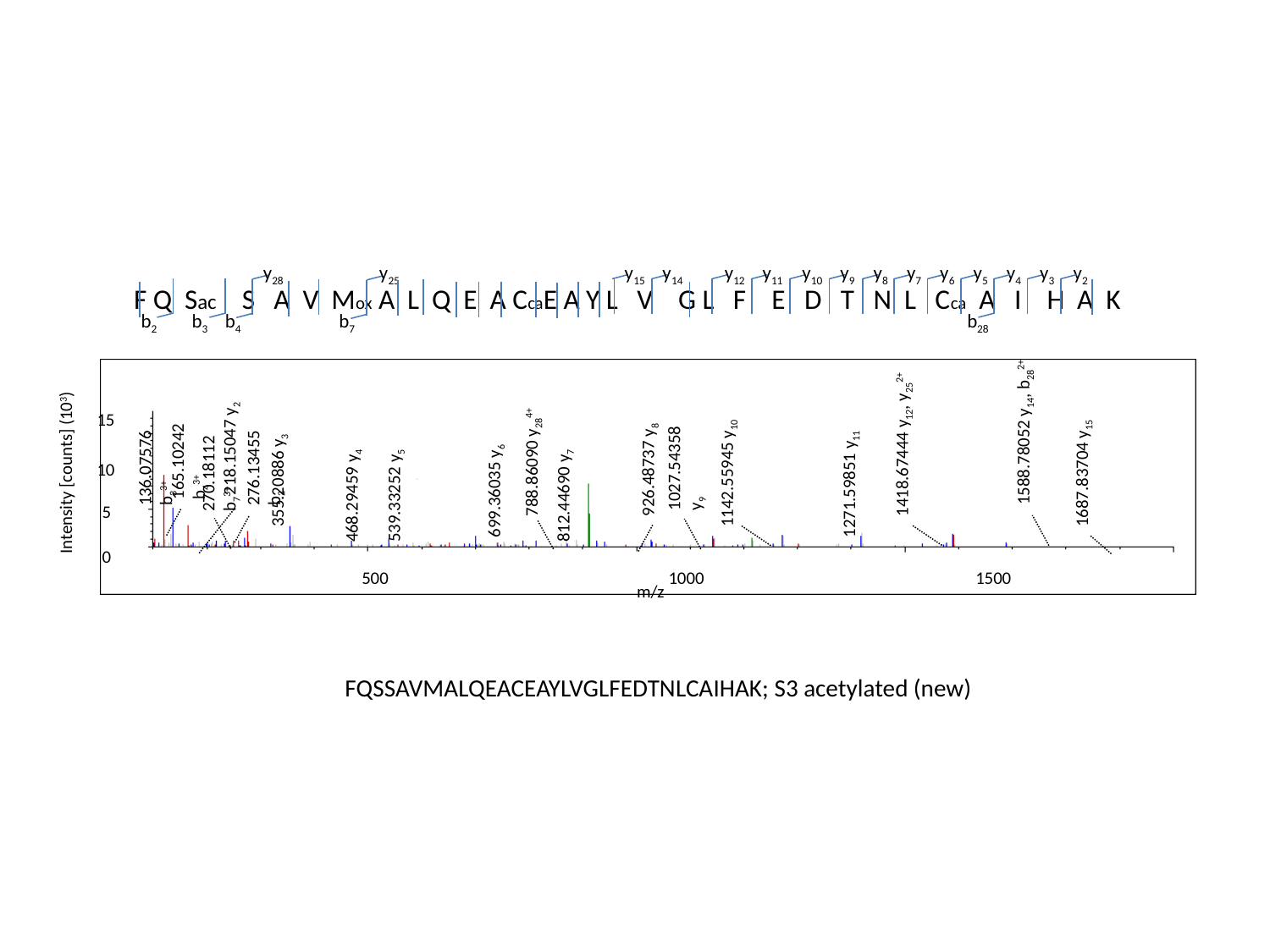

y28
y25
y15
y14
y12
y11
y10
y9
y8
y7
y6
y5
y4
y3
y2
F Q Sac S A V Mox A L Q E A CcaE A Y L V G L F E D T N L Cca A I H A K
b2
b3
b4
b7
b28
1588.78052 y14, b282+
788.86090 y284+
1418.67444 y12, y252+
Intensity [counts] (103)
165.10242 b43+
218.15047 y2
136.07576 b33+
270.18112 b73+
15
1027.54358 y9
276.13455 b2
926.48737 y8
1142.55945 y10
1687.83704 y15
355.20886 y3
1271.59851 y11
699.36035 y6
468.29459 y4
539.33252 y5
812.44690 y7
10
5
0
500
1000
1500
m/z
FQSSAVMALQEACEAYLVGLFEDTNLCAIHAK; S3 acetylated (new)

## Slide 6
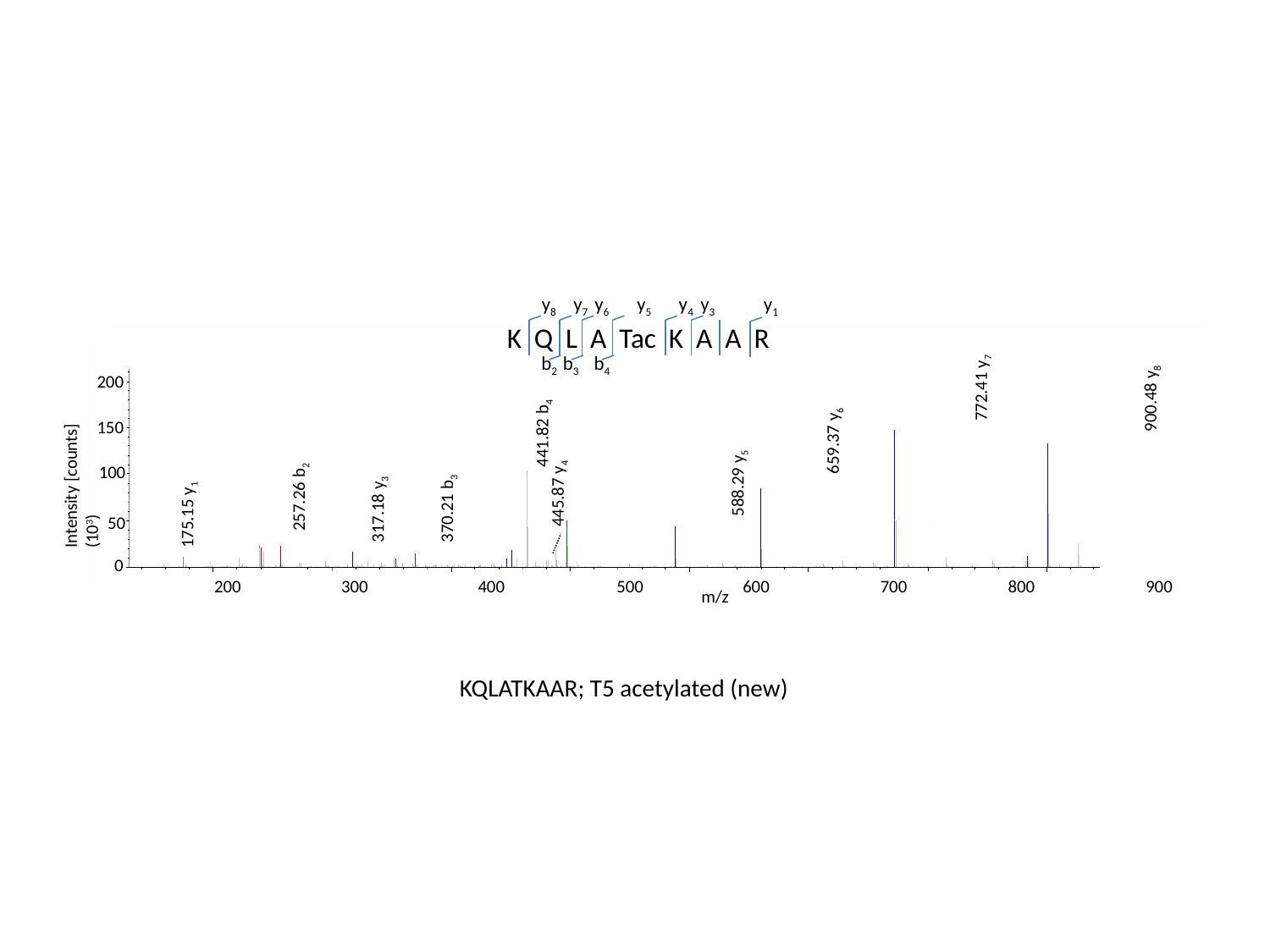

y8
y7
y6
y3
y1
b2
b4
 K Q L A Tac K A A R
y5
y4
b3
772.41 y7
900.48 y8
441.82 b4
200
50
0
659.37 y6
Intensity [counts] (103)
150
588.29 y5
445.87 y4
257.26 b2
317.18 y3
370.21 b3
175.15 y1
100
200
300
400
500
600
700
m/z
800
900
KQLATKAAR; T5 acetylated (new)

## Slide 7
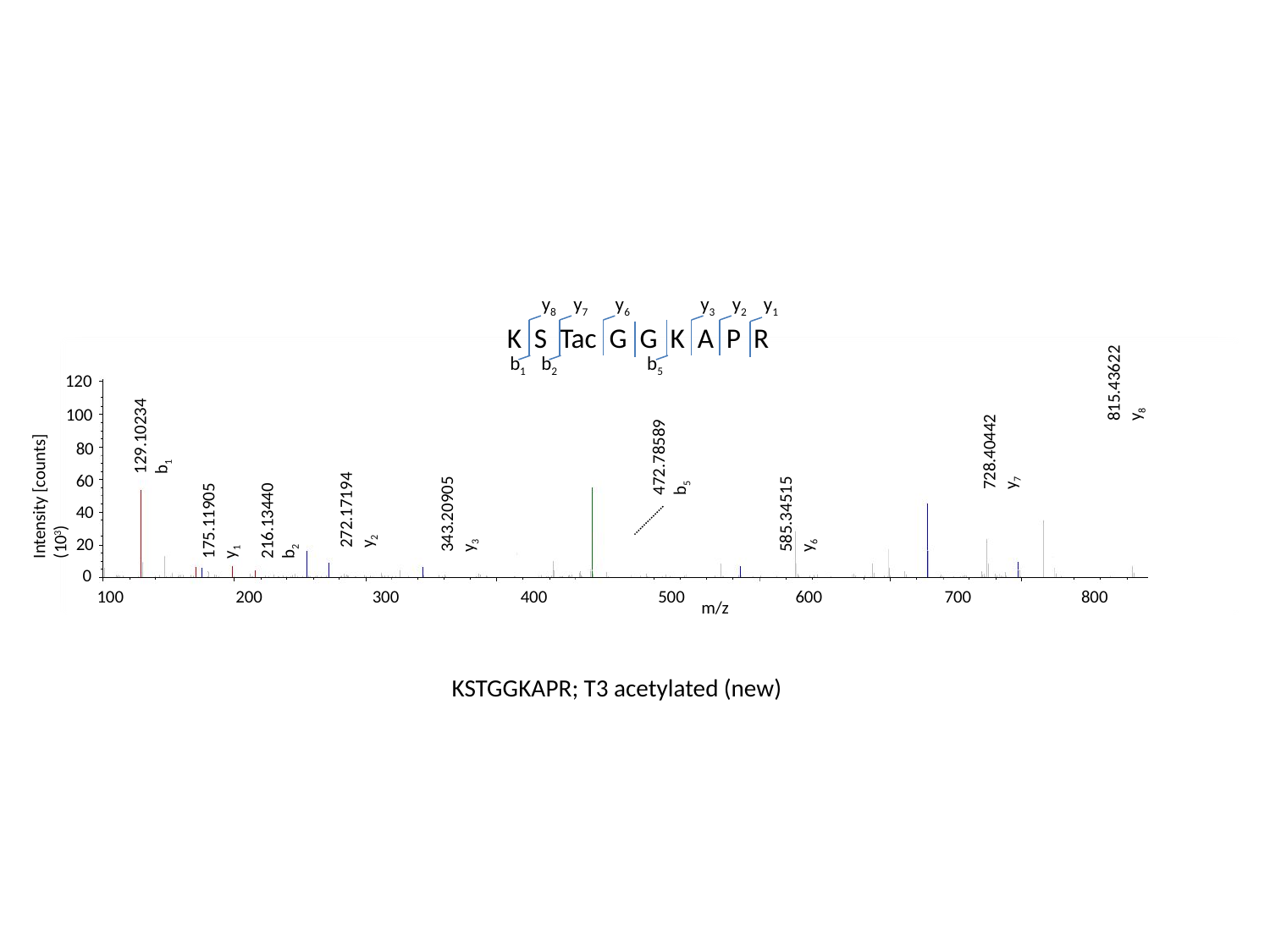

y8
y7
y6
y3
y2
y1
 K S Tac G G K A P R
815.43622 y8
b1
b2
b5
120
20
0
129.10234 b1
728.40442 y7
Intensity [counts] (103)
472.78589 b5
100
80
272.17194 y2
343.20905 y3
585.34515 y6
216.13440 b2
175.11905 y1
60
40
100
200
300
400
500
600
700
m/z
800
KSTGGKAPR; T3 acetylated (new)
